# Efficacy of Intrapleural or Intrapericardial Injection of Single Bevacizumab in the Treatment of Lung Cancer-Mediated Malignant Effusion

**DOI:** 10.1155/2022/6763625

**Published:** 2022-10-31

**Authors:** Dongyun He, Zhihua Guo, Zixian Xie, Yalei Zhang, Qiuhua Deng, Haihong Yang

**Affiliations:** ^1^Department of Thoracic Oncology, The First Affiliated Hospital, Guangzhou Medical University, State Key Laboratory of Respiratory Diseases, National Clinical Research Center of Respiratory Disease, Guangzhou, China; ^2^The Center for Translational Medicine, The First Affiliated Hospital, Guangzhou Medical University, Guangzhou, China

## Abstract

The usage of bevacizumab for malignant pleural effusion (MPE) or malignant pericardial effusion (MPCE) has attracted increasing interest from researchers, but the precise ways of bevacizumab administration remain unknown. Patients with histologically or cytologically confirmed non-small-cell lung cancer (NSCLC) with MPE or MPCE were enrolled in the study and treated with a low dose of single bevacizumab (100 mg) intrapleurally or intrapericardially injected after the drainage of the effusions. The Lung Cancer Symptom Scale (LCSS), efficacy, and safety of drug administration were used as evaluation parameters in this study. The results indicated that lung cancer-related symptoms were significantly improved following treatment, compared with symptoms before the treatment (LCSS, score 494 ± 78 vs. score 377 ± 77, mean ± SD) (*P* < 0.001). Malignant effusions were well controlled, and the median time to progression (TTP) was 91 days and 111 days in MPE and MPCE, respectively. In addition, no severe side effects were observed, except in one patient with mild dizziness. In summary, the low dose of single bevacizumab (100 mg) with intrapleural or intrapericardial injection is effective and safe in the treatment of lung cancer-mediated malignant effusion, rapidly improving the malignant effusion-related symptoms and quality of life in patients with NSCLC.

## 1. Introduction

Non-small-cell lung cancer (NSCLC) accounts for approximately 80% of lung cancer cases, the majority of which are already unresectable and metastatic at the time of initial diagnosis. In addition, approximately 40% of patients with advanced NSCLC will present with or develop malignant effusions (MEs), including pleural or pericardial effusion (MPE or MPCE) [1]. Potential symptoms of abnormal fluid accumulation in the pleural or pericardial space include cough, chest distress, dyspnea, and even right heart failure in patients with MPCE, threatening the survival of the patient and resulting in a significant decline in the quality of life (QOL) of the patients [2–5]. In addition, uncontrolled MPE or MPCE can affect the candidacy of patients to receive potentially life-extending anticancer therapies. One of the highest priorities in these patients is actively managing the MPE or MPCE in an attempt to improve the QOL [2, 6]. Currently, drainage of MPE or MPCE, thoracic or pericardial perfusion of chemotherapeutic drugs, and systemic chemotherapy are the primary means of handling MEs, while clinical pleurodesis is considered as a standard procedure for treating MPE but not MPCE [7]. These treatments have some effect on the tumor and provide relief from symptoms. However, patients treated with chemotherapy are vulnerable to bone marrow suppression and gastrointestinal side effects, and those who have received sclerosing agents can present with severe local side effects and be disqualified from intrapericardial injection. In addition, the repeated process of intercostal tube insertion can cause complications, including hemorrhage, organ injury, and infection [5].

MEs are associated with high levels of vascular endothelial growth factor (VEGF) in the serum or plasma [8–10]. VEGF plays an important role in the formation of MEs and one of the mechanisms is to increase vascular and mesothelial permeability and capillary fluid leakage [11, 12]. By blocking the binding of VEGF-A to its receptor, bevacizumab (BEV) induces the degradation of existing tumor blood vessels and normalization of the remaining blood vessels [13]. BEV can also substantially suppress the continuous growth and metastasis of tumor cells, by repressing angiogenesis in the tumor tissues [14]. The role of BEV in malignant progression suppression of pleural effusion has only recently been revealed and suggests that treatment with BEV, either as monotherapy or in combination with other chemotherapeutic agents, could be useful in the treatment of MPE [8, 15]. In addition, preclinical and clinical studies, although with several limitations, have demonstrated that inhibition of VEGF by BEV may represent an effective way to prevent local fluid accumulation. To date, the application of intrapleural or intrapericardial BEV therapy to MEs as a consequence of NSCLC, including appropriate dose and monotherapy or combination therapy, has not been investigated. Here, a prospective phase II trial of a low dose of single BEV in patients with advanced NSCLC with MPE or MPCE was conducted.

## 2. Patients and Methods

This prospective, open-labeled, single-arm, phase II clinical trial was performed at the First Affiliated Hospital of Guangzhou Medical University, China. All patients provided written informed consent prior to study-specific procedures. This study is registered with the Clinical Trail.gov Protocol Registration and Results System (PRS), under number NCT02054052.

### 2.1. Patient Selection

#### 2.1.1. Inclusion Criteria

1. Histological or cytological diagnosis of NSCLC
2. Cytological diagnosis of MPE or MPCE
3. Symptomatic MPE or MPCE evaluated by researchers
4. Unsuitable for or rejection of systemic therapy of tumor
5. Continuous tyrosine kinase inhibitors (TKI) treatment after TKI resistance, which is caused by MPE or MPCE
6. Estimated survival of more than 3 months
7. 18 years or older

#### 2.1.2. Exclusion Criteria

1. Current or recent (i.e., within 10 days prior to treatment) use of the full amount of inhibition of platelet function, anticoagulants, or thrombolytic therapy, which allows prophylactic anticoagulants
2. Allergy to BEV
3. Pregnant or lactating woman
4. Pleural or pericardial infection

### 2.2. Treatment Methods

After drainage of the effusions, single BEV (100 mg) dissolved in 50 ml of 0.9% normal saline was infused intrapleurally or intrapericardially. The chest tube was then removed.

### 2.3. Assessment Procedures

All patients underwent comprehensive baseline assessments including the lung cancer symptom scale (LCSS) [16, 17] and imaging studies. LCSS and chest radiography or ultrasonography were used to evaluate the therapeutic efficacy 21–30 days after the treatment. LCSS and chest radiography or ultrasonography were performed at least every two months in order to monitor the condition of the controlled MPE or MPCE. The response and duration of the response were determined by physicians and then recorded.

The therapeutic efficacy of BEV for MPE was evaluated according to the 1980 WHO standard for unified evaluation of efficacy: (1) complete remission (CR), i.e., effusion disappeared and maintained for at least 4 weeks; (2) partial remission (PR), i.e., effusion decreased significantly, at least 50% and maintained for more than 4 weeks; (3) ineffective (NC), i.e., the effusion decreased by less than 50% or increased but not more than 25%; and (4) progress (PD), i.e., the effusion increased significantly or the patient died. The therapeutic efficacy of BEV for MPCE was classified as follows, according to previous studies [18–20] and the Response Evaluation Criteria in Solid Tumors (RECIST, version 1.1): (1) CR, i.e., pericardial effusion completely disappeared within 4 weeks; (2) PR, i.e., pericardial effusion was reduced by more than 50% within 4 weeks; (3) NR, i.e., pericardial effusion was reduced by less than 50% or effusion increased; and (4) PD, i.e., a progression of effusion was defined as when MPE or MPCE of more than 25% of the long axis of the hemithorax or 50% of the pericardial cavity was observed or tube drainage was needed. Toxicity evaluations were based on the Common Terminology Criteria for Adverse Events, version 4.0. The relationship of each event to treatment was assessed by a physician and then recorded. Adverse events (AEs) were recorded for up to one month after the treatment of MPE or MPCE.

### 2.4. Study Design and Statistical Analysis

This trial was a prospective, single-arm, open-label phase II trial aiming to examine the efficacy and safety of BEV intrapleural or intrapericardial injection in the treatment of lung cancer-mediated malignant effusion. The primary endpoint was the LCSS estimation. The secondary endpoint was the overall response rate (ORR), including CR and PR, time to progression (TTP), and number of participants with AEs. ORR was defined as an unequivocal reduction 21–30 days after the treatment, compared to baseline MEs on a chest radiograph or ultrasonography. TTP was defined as the time from enrollment to the date of confirmation of progression in MEs, or death from any cause. Kaplan–Meier (K-M) plots were used for TTP analyses, and the median and 95% CI were determined. Statistical significance was defined at the 0.05 level. Statistical analysis was performed using SPSS 16.0 software. The cutoff date was March 27, 2019.

## 3. Results

### 3.1. Patients and Treatment Characteristics

A total of 22 patients were enrolled between January 2014 and March 2019 in the study. Patient characteristics are summarized in Table 1. The majority of patients (18/22, 78.3%) had an Eastern Cooperative Oncology Group-performance status (ECOG-PS) of two or three. The median age was 63 years (range 43–86 years). 20 patients had lung adenocarcinoma, two patients had lung squamous cell carcinoma, and 20 patients (91%) received non-first-line treatment.

### 3.2. Adverse Events

The patients exhibited no severe side effects, except one patient with mild dizziness.

### 3.3. Clinical Outcome

The symptoms of NSCLC were clearly relieved after local treatment of MEs. LCSS after the treatment (score 494 ± 78, mean ± SD) was significantly improved compared with the score before the treatment (score 377 ± 77, mean ± SD) (paired differences: score 117 ± 64, mean ± SD, 95% CI: score 89–145). In addition, the performance status (PS) score (paired differences: score −1.3 ± 0.8, mean ± SD, 95% CI: score −1.7 to −1) improved significantly. The detailed data, including MPE and MPCE, are shown in Table 1.

The effusions clearly decreased three weeks after the treatment compared with those before the treatment. The ORR of MEs for single BEV treatment was 86.4%, where eleven MPE and eight MPCE patients achieved PR, two MPE patients achieved NR, and one patient was unable to be evaluated (Table 1). In six of the patients, the effusions did not increase before death, and the survival time from BEV treatment to death ranged from 22 to 224 days (median, 168 days). Only one patient experienced a recurrence of effusion before death. Good effects were presented in 20 patients with hemorrhagic effusion, while poor effects were presented in 2 patients with nonhemorrhagic pleural effusion.

**Figure 1:**
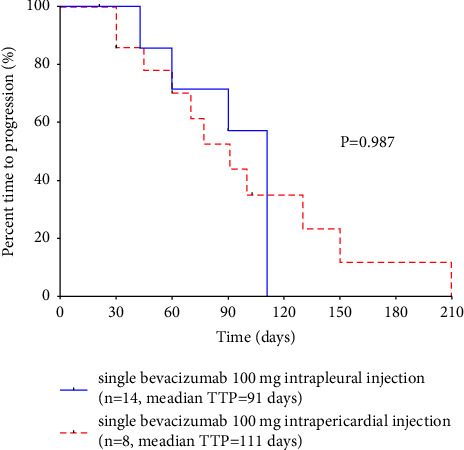
Time to progression of patients with single bevacizumab (100 mg) intrapleural or intrapericardial injection.

The TTP of MPE was 91 days, while the TTP was 111 days in MPCE. No significant difference was detected in the remission time between MPE and MPCE (*P* = 0.987) (Figure 1).

## 4. Discussion

The patients with MPE or MPCE that were enrolled in the study had squamous cell carcinoma and non-squamous cell carcinoma. Although the systemic indication for BEV is non-squamous cell carcinoma, MEs caused by squamous cell carcinoma were also safe to be included in the study with high tolerance and without any severe side effects. Underlying mechanisms may result from that in NSCLC patients, the reabsorption of pleural or pericardial fluid is blocked due to vascular or lymph node metastasis. By contrast, cancer cells, regardless of type, secrete various growth factors, which could lead to the local overexpression of VEGF and thus contribute to elevated capillary permeability and fluid release from the capillary beds [8, 12, 21–23].

During this study, no severe side effects were observed in the patients, indicating that this line of therapy is highly tolerated. The single BEV therapy used in our study was able to control MPE or MPCE within a short period of time. No withdrawal of subjects from our study was recorded due to toxicity. Although mild dizziness appeared in one patient, it resolved spontaneously and quickly without further medication. Furthermore, no hypertension, proteinuria, thrombosis, gastrointestinal, or pulmonary bleeding were observed. The results indicate that treatment with intrapleural or intracardial delivery of low-dose single BEV therapy is reliable, safe, and feasible, providing a possible novel approach for the management of MEs, without impeding subsequent systemic treatment due to side effects.

Our study provided evidence that low-dose therapy with intrapleural or intracardial single BEV can efficiently treat MPE or MPCE caused by NSCLC. This single therapy can quickly benefit the primary outcome in patients, by which MEs-related clinical symptoms rapidly relieved and patient physical status and QOL significantly improved. Compared with the previous courses of treatment, a single dose of BEV administration showed low or no side effects and improved LCSS, which provides a good opportunity for lung cancer patients to undergo systematic therapy to have a better response rate and survival rate [24]. In terms of the efficacy of cancer treatment, QOL is considered one of the key indicators. With the same survival time, a treatment that can significantly improve QOL is considered to be highly beneficial to patients [25]. Compared with chemotherapy drugs such as platinum alone, intrapleural or intracardial injection of a single BEV reduced the incidence of chest pain and mitigated the dyspnea of patients with MEs, indicating the improvement of the QOL of patients with MEs.

This study showed that low-dose single BEV in the treatment of lung cancer-mediated MEs seems to favor a modest improvement in the secondary outcomes, ORR and TTP. The local injection proved the ability to decrease and control effusion rapidly for a relatively long time with no further systemic therapy. No difference between MPE or MPCE in TTP time was observed. Two patients with yellow effusion were recorded who had less effective BEV therapy, compared with that of other patients with red effusion. Previous studies have shown that overexpression of VEGF in MEs is associated with red effusions and on contrast, in yellow effusions [11, 26]. Thus, in the case of the recurrence of MEs, we can repeatedly inject a single dose of BEV to ease the symptoms because it is safe and easy to operate with mild toxicity and endurance [27]. Therefore, we can quickly control ME-associated symptoms and improve QOL from the perspective of palliative care.

Although low-dose single BEV therapy and thoracic hyperthermia treatment of MPE can both be quickly effective for patients and control the MPE well, pleural heat perfusion is much more expensive than single BEV and patients may experience severe chest pain. Based on the lack of an effective and safe local treatment for MPCE, low-dose single BEV therapy may stand as an excellent choice as a cost-effective MPCE treatment option.

## 5. Conclusion

Intrapleural or intracardial single low-dose BEV therapy may represent a novel treatment option for malignant pleural effusion or malignant pericardial effusion caused by non-small-cell lung cancer. It is effective and safe in the treatment of lung cancer-mediated malignant effusions, laying the foundation for subsequent systemic treatment.

## Figures and Tables

**Table 1:** Patient characteristics.

Characteristics	Local injection site	
	Intrapleural (%)	Intrapericardial (%)
Age (years)	*n* = 14	*n* = 8
<65	6 (42.9)	0
≥65	8 (57.1)	8 (100)

Sex	*n* = 14	*n* = 8
Male	9 (64.3)	5 (62.5)
Female	5 (35.7)	3 (37.5)

Pathology	*n* = 14	*n* = 8
Adenocarcinoma	14 (100)	6 (75)
Squamous	0	2 (25)

Lines of treatment	*n* = 14	*n* = 8
First-line	1 (7.1)	1 (12.5)
Non-first-line	13 (92.9)	7 (87.5)

LCSS (mean ± SD)	*n* = 14	*n* = 8
Before treatment (days)	127 ± 41	91 ± 12
After treatment (days)		

PS (mean ± SD)	*n* = 14	*n* = 8
Before treatment	2.5 ± 0.65	3 ± 0.93
After treatment	1.3 ± 0.91	1.4 ± 0.92

Adverse effect	*n* = 14	*n* = 8
Dizzy	1 (7.1)	0

## Data Availability

This study is registered with the Clinical Trail.gov Protocol Registration and Results System (PRS), under number NCT02054052.
